# Current status of research regarding *Blastocystis* sp., an enigmatic protist, in Brazil

**DOI:** 10.6061/clinics/2021/e2489

**Published:** 2021-06-29

**Authors:** Gessica Baptista de Melo, Larissa Rodrigues Bosqui, Idessania Nazareth da Costa, Fabiana Martins de Paula, Ronaldo Cesar Borges Gryschek

**Affiliations:** I Laboratorio de Investigacao Medica (LIM06-Laboratorio de Imunopatologia da Esquistossome), Hospital das Clinicas HCFMUSP, Faculdade de Medicina, Universidade de Sao Paulo, Sao Paulo, SP, BR.; II Secao de Helmintologia, Instituto de Medicina Tropical (IMT), Faculdade de Medicina FMUSP, Universidade de Sao Paulo, Sao Paulo, SP, BR.; III Laboratorio de Imunoparasitologia das Doencas Negligenciadas e Cancer, Universidade Estadual de Londrina (UEL), Londrina, PR, BR.

**Keywords:** *Blastocystis* sp., Parasitological Diagnosis, Molecular Diagnosis, Brazil

## Abstract

The present study aimed to evaluate the occurrence of *Blastocystis* sp. in Brazilian studies over a period of years (2000-2020), as well as point out relevant aspects of this enigmatic organism.

We performed a literature search using six sources of international databases. The data were divided into diagnostic by parasitological and molecular techniques, and relevant aspects. After applying the inclusion and exclusion criteria, 52 studies were included in the final analysis.

The occurrence of *Blastocystis* sp. in Brazil ranged from 0.5% to 86.6%, as determined using parasitological techniques. The highest occurrence was in the North (27.3%) and the lowest, in the Midwest region (13.4%). In Brazil, most studies have employed molecular techniques and are concentrated in the Southeast region. The *Blastocystis* sp. subtype ST3 had the highest average positivity, followed by ST1 and ST2.

These findings represent a panorama that reflects the reality of Brazil; thus, we believe that the effectiveness of parasitological diagnosis should be considered with regard to making an appropriate choice of technique for detecting *Blastocystis* sp. Additionally, we emphasize the importance of further studies in the context of molecular epidemiology with regard to this genus. *Blastocystis* sp. is not well understood yet, and very little information regarding this genus is available; hence, further research regarding this genus is urgently needed.

## INTRODUCTION

It is believed that the organism we know today as *Blastocystis* sp. was discovered well before its first description in the literature (1). Throughout history, researchers have reported that hollow spherical cells are prevalent in fecal samples from humans and animals, very similar to artifacts and degraded cells. The first accepted description of *Blastocystis* sp. was published by Alexeieff (2), when cells from animal fecal isolates resembled the fungus of the genus *Blastomyces* (Greek ‘kystis’: cyst) (3). Even 100 years after this first description, the real clinical and biological importance of this enigmatic protist is poorly understood.

Currently, it is classified as a protist member of the phylum Stramenopiles (4,5). It is known that this organism has a worldwide distribution, infecting more than one billion individuals (6,7). *Blastocystis* is genetically diverse, with 17 subtypes (ST1-ST17) based on polymorphic regions of its small subunit of the ribosomal RNA gene (SSU-rDNA) (7-10). Some of these subtypes are found in different hosts, but others are exclusively found in humans (8,10). Humans serve as hosts for nine subtypes (ST1-ST9), with ST3 being the most prevalent, followed by ST1, ST2, and ST4 (7,9). Several studies have identified *Blastocystis* subtypes in Brazil. Among these, it was observed that the distribution of this genus in Brazil follows the world standard, with the high prevalence of ST3, ST1, and ST2, in addition to the occurrence of subtypes rarely found in humans, such as ST6, ST7, and ST8 (11-14).

In Brazil, the first specific study of *Blastocystis* sp. was carried out by Guimarães and Sogayar (15). In this study, the high positivity of this organism was demonstrated by parasitological methods in children and daycare center workers. Over the years, new studies have been developed with the main objective of detecting intestinal parasites, confirming the high positivity rate of *Blastocystis* sp. (11-19). However, the importance of this organism remains to be determined. The present study aimed to evaluate the occurrence of *Blastocystis* sp. in Brazilian studies over a period of 20 years (2000-2020), as well as point out some relevant aspects of this mysterious organism.

## METHODS

We conducted a comprehensive and descriptive literature search regarding the occurrence of *Blastocystis* sp. using parasitological and molecular techniques in Brazil. We performed a literature search in six international databases: US National Library Online (PubMed), Scientific Electronic Library Online (SciELO), Web of Science, Science Direct, Scopus, and Google Scholar, in order to obtain the maximum number of relevant studies for the scope of this review. The literature search process used the keywords terms: “parasite,” “intestinal parasite,” “*Blastocystis*” and “Brazil,” alone or in combination with “AND.” As inclusion criteria, we used original research papers or short communications reporting the occurrence of *Blastocystis* sp. from Brazil published online in English and Portuguese in the last 20 years (2000-2020). Studies published before 2000, those that did not report the occurrence of *Blastocystis* sp., and those that were not published in English or Portuguese were excluded.

Various characteristics, including first author, year of publication, region, state, population, diagnostic method, sample size, and positivity for *Blastocystis* sp. in each article included in this study were extracted and recorded using Excel software (Microsoft, Redmond, WA, USA). The data were divided into diagnostic by parasitological and molecular techniques, and relevant aspects. 

## RESULTS AND DISCUSSION

After applying the inclusion and exclusion criteria, 52 studies were included in the final analysis to estimate the occurrence and molecular identification of *Blastocystis* sp. in Brazil between 2000 and 2020.

### Detection of *Blastocystis* sp. by parasitological techniques

Considering the parasitological diagnosis, the number of studies that reported positivity for *Blastocystis* sp. varied between the different regions of Brazil (Table 1) (20-64), with the Southeast region contributing 26 studies, followed by the South region with 11, the North region with 7, the Midwest region with 5, and the Northeast with 3. It is worth mentioning that of the studies included, only nine had the specific objective of searching for *Blastocystis* sp. in fecal samples.

**Figure 1:**
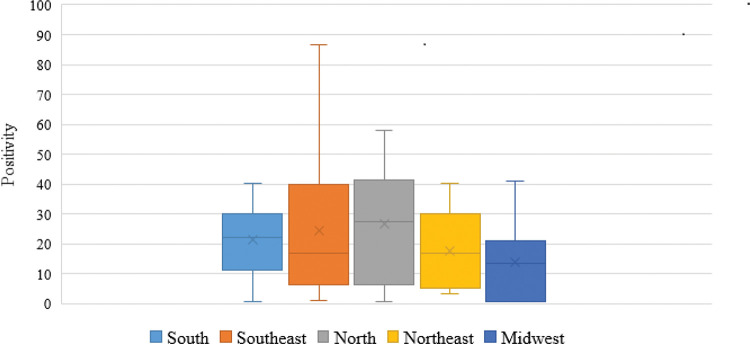
Occurrence of *Blastocystis* sp. in fecal samples in humans based on the parasitological methods used for their detection and the region in Brazil (2000-2020).

The occurrence of *Blastocystis* sp. in Brazil ranged from 0.5% to 86.6% (median, 23±21), as per the use of parasitological techniques (Table 1). It can be seen that the highest occurrence rate was in the north (27.3±22.4), followed by the southeast (24.5±23.8), south (22.2±13.7), northeast (16.9±20.2), and midwest regions (13.4±17.6) (Figure 1).

In general, several techniques are employed for parasitological diagnosis, such as direct examination, spontaneous sedimentation, concentration techniques on formaldehyde-ether, and permanently stained smears (8,29,65-67). Of the evaluated studies, the most commonly used concentration techniques were the spontaneous sedimentation method (33/52) or centrifugation sedimentation (37/52). It is noteworthy that methods that require the addition of water or a preservative solution, with a centrifugation step, can destroy the structure of this organism, resulting in false-negative results (68). It is observed that the positivity for *Blastocystis* sp., considering the concentration methods, was quite variable, from 0.5 to 63.1% (28,39,51). Some authors claim that preservative solutions and centrifugation can assist in obtaining *Blastocystis* forms, even facilitating their visualization (29).

It can be observed that the results for *Blastocystis* sp. detection differed in various studies, for example, in studies using the TF-Test kit for parasitological determination (18,30,31). These discrepancies may be due to differences in the regions and populations studied, since Brazil is a country with diverse regional differences, especially in terms of climate and socioeconomic factors.

The most appropriate methods for diagnosis are *in vitro* cultures and permanent-stained smears, which enable the better visualization of, and differentiation between, different *Blastocystis* sp. forms, but require more time for implementation (8,29,66,68-71). Of the studies evaluated, a few studies used permanent stained smears (19/52) and only two studies used *in vitro* cultures as diagnostic techniques. It was observed that the average *Blastocystis*-positivity rate (22.1±18.1) of these studies was not greater considering the average occurrence rate of this organism in Brazil. However, we can consider the best visualization of the forms, which considerably facilitates the identification of this organism. Nevertheless, recent studies (19,40) have shown similar results with regard to comparing the *in vitro* culture methods with the spontaneous sedimentation technique. It is interesting to note that in many of the studies evaluated herein, permanent stains were used to stain the sediments resulting from *in vitro* cultures (12,19), and not as an initial choice of technique after obtaining a smear from the fresh fecal sample.

The studies whose main objective was to evaluate the occurrence of *Blastocystis* in different fecal samples showed a positivity rate that ranged from 17.8 to 55.8% (Table 1). Of these, only two used permanently stained smears as a technique for parasitological diagnosis (19,49).

Important aspects related to the parasitological diagnosis of *Blastocystis* sp. First, it is known that the use of preservative solution in the fecal sample, associated with the choice of techniques for the detection of this parasite in routine laboratory settings, may result in an underestimation of the real number of positive samples. In addition, this method of diagnosis requires considerable technical expertise to recognize the different forms of this organism. Conversely, the high number of positive parasitological results for *Blastocystis* has attracted the attention of several researchers. This fact allows us to question whether this high number represents the real occurrence of *Blastocystis*, especially in view of the difficulty of its morphological identification (6). It also allows us to question the implications of this high positivity rate.

The great diversity of the collected data leads to a discussion and superficial analysis of the results. Moreover, it is noteworthy that most studies aimed to evaluate the overall occurrence of intestinal parasites, which makes the effective determination of its real prevalence difficult, especially with regard to the techniques of choice for the parasitological diagnosis of *Blastocystis* sp. 

### Detection of *Blastocystis* sp. by molecular techniques

We must emphasize that in most studies carried out in Brazil and cited here, molecular techniques were not used for molecular diagnosis, but instead, as tools for identifying *Blastocystis* subtypes present in samples that were previously reported to be positive for *Blastocystis* sp. based on parasitological techniques. The amplification of specific DNA from stool samples has allowed for new perspectives on the laboratory diagnosis of *Blastocystis* sp., mainly due to the high sensitivity and specificity of molecular techniques (6). However, the use of molecular techniques in all studies is sometimes difficult because of their high cost.

Till date, in Brazil, only 10 studies have employed molecular techniques and these are concentrated in the southeast region. Of these, only Silva et al. (72) reported the use of PCR as a diagnostic method (16.0%) for *Blastocystis* sp. in fecal samples from transplant candidates. Other studies used PCR only after confirmation of the positive parasitological diagnosis, with the aim of the molecular characterization of *Blastocystis* subtypes in the samples.

Molecular methods are commonly used to identify and determine the distribution of different subtypes of *Blastocystis* sp. (73). As a result, aspects that remain unknown, especially, epidemiological and pathological characteristics, have been noted. It is currently known that the genus *Blastocystis* is composed of 17 subtypes (ST1-ST17) that can infect human and non-human hosts (9,10). We highlight that the first study characterizing *Blastocystis* subtypes carried out in Brazil was by Malheiros et al. (11), who analyzed the fecal samples of indigenous people from the Mato Grosso State.

The distribution of *Blastocystis* subtypes in Brazil is shown in Table 2 (11-14,19,20,40,72,74,75); ST3 showed the highest average positivity rate (38.5±11.4), followed by ST1 (35.0±9.1) and ST2 (16.2±8.4). These data confirm the findings from previously published international literature, which shows that more than 90% of isolates in humans belong to four subtypes (ST1, ST2, ST3, and ST4) (7,9), while the others occur more frequently in animals (7,8). Mixed infections were demonstrated in five studies, which showed an average positivity rate of 3.4%±4.9. Several reports in the current literature indicate the occurrence of mixed infections.

Different primers have been described for the detection of *Blastocystis* sp. DNA, considering the analysis of *Blastocystis* SSU-rDNA. Initially, specific primers were used for each subtype (76,77), followed by a primer-called pan-*Blastocystis* barcode (78). Although this barcode region is the most used, other initiators have been proposed, especially in several studies carried out in Brazil (19,40). Harmonization of the current nomenclature used in this field has been proposed to facilitate the comparison and characterization of various *Blastocystis* subtypes in different studies (10).

With the growing number of studies, *Blastocystis* subtypes that had not been described in Brazil have recently been identified. In this context, we can mention that ST4, which was considered to be restricted to Europe (79), has recently been described in four studies in Brazil (14,19,40,74); ST4 has been associated with the presence of symptoms of *Blastocystis* infection (79). In addition, ST7 (12,72,75) and ST8 (14,19) have been reported in the southeast and southern regions of Brazil.

Although molecular PCR has clearly enabled further improvements in diagnostic and epidemiological studies, its successful application depends on understanding the limitations and assumptions associated with its use (31). In this regard, DNA extraction from fecal samples, the choice of a suitable primer, and obtaining viable sequences should be considered important limitations associated with the use of PCR-based methods (80). 

Although the subtypes of *Blastocystis* sp. present great genetic variability, it is not known whether this can influence factors related to the host (70). This variability has been demonstrated in studies conducted in Brazil and may explain the pathology of this organism (8,67,73). Among the studies evaluated, only a few addressed the pathological aspects related to the presence of this organism. Melo et al. 2019 (74) reported the occurrence of *Blastocystis* sp. in patients with chronic urticaria; however, the absence of a control group compromises the conclusions drawn.

It is important to highlight the recent approach followed in *Blastocystis*-related studies with regard to its presence in the intestinal microbiota and the possible pathological implications of this phenomenon (10,81). However, no study in Brazil has evaluated this issue yet.

## RELEVANT ASPECTS

The fecal-oral route of transmission and its survival capacity in different organisms, such as humans and animals, likely explains the global distribution of *Blastocystis* (10). In addition, the American continent is now considered to have ideal conditions for the high prevalence of this organism, such as increased rates of poverty, inadequate sanitation, and lack of potable water in many regions (10).

It is common to evaluate different environmental samples with the expectation of a transmission link between intestinal parasites and the environment. In studies carried out in Brazil, the high positivity rate of *Blastocystis* in natural water sources (82) and in plants sold in markets (83) was noted. This reinforces the need for monitoring parasite contamination and the importance of educational campaigns.

Another pertinent issue when considering this organism is the possibility of zoonotic transmission. Research reinforces the need for the investigation of *Blastocystis* sp. in samples of wild and domestic animals (84,85). Data concerning the occurrence of its subtypes in humans and animals in Brazil are limited. From the evaluated studies, it was possible to identify ST1-5 and ST8 in samples from non-human hosts (40,75,86). We believe that new studies can determine the origin of these subtypes and the relationship between humans and animals with regard to harboring this organism.

An interesting point to address is the different epidemiological patterns of the colonization and/or infection of this organism. Although there is evidence that some subtypes may be related to clinical manifestations, the studies presented in Brazil are still inconclusive (74), mainly due to the high proportion of asymptomatic carriers (12,72). Information regarding the variation in the distribution of subtypes in Brazil has only just begun to emerge, and there are still major gaps to be filled. Thus, new studies on the molecular epidemiology of *Blastocystis* can help improve the knowledge about the pathogenicity of its infection.

## CONCLUSION

The history of research regarding *Blastocystis* sp. exposes much uncertainty regarding its biological and pathogenic aspects. This observation reflects the reality of Brazil; thus, we believe that the effectiveness of parasitological diagnosis should be considered with regard to making an appropriate choice of technique for detecting *Blastocystis* sp. Thus, based on our review, we suggest the use of fresh fecal smears, followed by permanent staining, to increase the effectiveness of the results. It should be remembered that Brazil is a country with wide regional and dimensional variations, in addition to socioeconomic factors that can directly affect the positivity rate of *Blastocystis*.

Additionally, we emphasize the importance of further studies in the context of molecular epidemiology, particularly in increasing knowledge about the genetic diversity of *Blastocystis* sp. This would improve the elucidation of the biological and pathological aspects regarding this genus that remain unclear. *Blastocystis* sp. is an important organism, and further studies will work toward answering the many questions that studies regarding this genus have brought about so far.

## AUTHOR CONTRIBUTIONS

Melo GB and Paula FM contributed to the conception and design of the study. Melo GB and Bosqui LR performed the literature search. Melo GB and Paula FM wrote the manuscript. Melo GB, Paula FM, Bosqui LR, Costa IN and Gryschek RCB reviewed and approved the final version of the manuscript.

## Figures and Tables

**Table 1:** Occurrence of *Blastocystis* sp. in fecal samples in humans based on the parasitological methods used, the region and state in Brazil, and the population studied (2000-2020).

Region	State	Population	Parasitological techniques	No. positive %	Reference
South	PR	Children	R, PS	8.9	Oishi et al. (20)
		All	R, KK, PS	28.2	Seguí et al. (14)
		Children	R	31.8	Seguí et al. (21)
		All	SS, R, PS	28.2	Takizawa et al. (22)
		Adult	SS, BM, F, PS	20.9	Kulik et al. (23)
		All	D, R, F, PS	26.5	Nascimento and Moitinho (24)
	SC	Children	SS, F, PS	40.4	Santos et al. (25)
		Children	D, SS, R	4.0	Batista et al. (26)
		Adult	SS, BM, F, G	14.3	Nolla et al. (27)
		Children	SS, F	0.5	Quadros et al. (28)
	RS	ND	SS, R, PS	40.0	Eymael et al. (29)
Southeast	SP	Children	TF	69.6	Santos et al. (30)
		All	TF	66.6-86.6	Rebolla et al. (18)
		All	D, F, PS	7.9	David et al. (12)
		Children	F, TF, PS	1.2	David et al. (30)
		All	D, SS, BM, F, PS	4.6	Miné and Rosa (32)
		All	R, BM, KK, PS	12.1	Anaruma Filho et al. (33)
		All	D, BM, F, KK	16.7	Martins et al. (34)
		Children	SS, R, F, PS	14.3	Carvalho et al. (35)
		Children	SS, R, PS	1.6	Carvalho-Almeida et al. (36)
		Children	D, SS, F	38.3	Amato Neto et al. (37)
	RJ	All	SS, F, IC	55.8	Barbosa et al. (19)
		Adult	SS, BM	41.2-45.2	Gama et al. (38)
		All	BM, F, KK	12.7	Faria et al. (39)
		All	SS, IC	27.0-35.5	Valença-Barbosa et al. (40)
		Children	SS, R, BM, F	3.8	Torres de Freitas et al. (41)
		All	SS, BM	6.7	Macedo et al. (42)
		All	R, PS	4.4-8.7	Silva-Neto et al. (43)
		All	SS, R, BM, F	6.1	Uchôa et al. (44)
		Children	D, R, PS	1.4	Carvalho-Costa et al. (45)
		Children	R	7.6	Pinheiro et al. (46)
		Adult	SS, BM, F	12.1	Port Lourenço et al. (47)
		All	SS, BM, F, PS	1.1	Uchoâ et al. (48)
	MG	All	D, R, PS	17.8	Cabrine-Santos et al. (49)
		Adult	R	24.5-41.9	Gil et al. (17)
		All	R	22.4	Gil et al. (50)
		All	R, KK	63.1	Martins et al. (51)
Midwest	MS	Adult	SS, R	3.9	Curval et al. (52)
		All	SS	40.9	Aguiar et al. (53)
	MT	Children	SS, PS	0.5	Luz et al. (54)
		All	SS, R	21.0	Malheiros et al. (11)
	GO	Adult	SS, BM, F, PS	0.5	Souza Junior et al. (55)
North	AM	All	SS, TF	43.4	Gonçalves et al. (56)
		All	SS	0.7	Visser et al. (57)
		All	R, BM, KK, PS	39.1	Carvalho-Costa et al. (58)
	PA	All	SS, KK	5.2	Cardoso et al. (59)
		All	D, SS	37.3	Loureiro et al. (60)
		All	D, SS, F, PS	57.8	Borges et al. (16)
	RO	All	R, F	7.7	Palhano-Silva et al. (61)
Northeast	SE	Children	R	40.1	Oliveira et al. (62)
	PE	Adult	R, BM, PS	7.3	Arcoverde et al. (63)
	PI	All	SS	3.4	Alves et al. (64)

ND, no date; parasitological techniques, D, direct method; SS, spontaneous sedimentation method; R, centrifugation sedimentation; BM, Baermann-Moraes method; F, flotation concentration; KK, Kato-Katz method; IC, *in vitro* culture; TF, TF-test kit; HM, Harada-Mori method; G, Graham method; PS, permanent-stained smears. PR, Paraná; SC, Santa Catarina; RS, Rio Grande do Sul; SP, São Paulo; RJ, Rio de Janeiro; MG, Minas Gerais; MS, Mato Grosso do Sul; MT, Mato Grosso; GO, Goiás; AM, Amazonas; PA, Pará; RO, Rondonia; SE, Sergipe; PE, Pernambuco; PI, Piauí.

**Table 2:** Occurrence of the subtypes of *Blastocystis* sp. human fecal samples based on the region and state in Brazil and the primers used.

Region	State	Primers	Subtypes (%)											Reference
			ST1	ST2	ST3	ST4	ST5	ST6	ST7	ST8	ST9	mixed STs	non-identified ST	
South	PR	pan-*Blastocystis* barcode	36.4	21.2	39.4	-	-	-	-	-	-	3.0	-	Oishi et al. (20)
		pan-*Blastocystis* barcode	36.3	15.7	41.2	2.9	-	1.0	-	2.9	-	-	-	Seguí et al. (14)
Southest	SP	pan-*Blastocystis* barcode	37.5	12.5	45.8	-	-	-	4.2	-	-	-	-	Silva et al. (72)
		pan-Blastocystis barcode	25.0	17.8	28.5	21.4	-	3.6	-	-	-	3.6	-	Melo et al. (74)
		pan-Blastocystis barcode	54.4	7.0	33.3	-	-	-	5.3	-	-	-	-	Oliveira-Arbex et al. (75)
		pan-*Blastocystis* barcode	22.5	12.5	60.0	-	-	5.0	-	-	-	-	-	Melo et al. (13)
		pan-Blastocystis barcode	34.3	10.5	43.2	-	-	3.0	6.0	-	-	3.0	-	David et al. (12)
	RJ	Blast 505-532/Blast 998-1017	27.0	27.0	34.0	3.5	-	-	-	7.0	-	-	1.17	Barbosa et al. (19)
		Blast 505-532/Blast 998-1017	35.9	6.2	42.2	1.6	-	-	-	-	-	14.1	-	Valença-Barbosa et al. (40)
Midwest	MT	pan-Blastocystis barcode	41.0	32.0	17.0	-	-	-	-	-	-	10.0	-	Malheiros et al. (11)

PR, Paraná; SP, São Paulo; RJ, Rio de Janeiro; MT, Mato Grosso.
